# The Role of Antioxidants in the Treatment of Metabolic Dysfunction-Associated Fatty Liver Disease: A Systematic Review

**DOI:** 10.3390/antiox13070797

**Published:** 2024-06-29

**Authors:** Kiana Mohammadian, Fatemeh Fakhar, Shayan Keramat, Agata Stanek

**Affiliations:** 1 Division of Hematology and Blood Banking, Department of Medical Laboratory Sciences, School of Paramedical Sciences, Shiraz University of Medical Sciences, Shiraz 71348, Iran; kiana.mohammadian78@gmail.com (K.M.); fatemehfakhar1999@gmail.com (F.F.); 2 VAS-European Independent Foundation in Angiology/Vascular Medicine, Via GB Grassi 74, 20157 Milan, Italy; shayan.sk1993@gmail.com; 3 Support Association of Patients of Buerger’s Disease, Buerger’s Disease NGO, Mashhad 9183785195, Iran; 4 Department and Clinic of Internal Medicine, Angiology, and Physical Medicine, Faculty of Medical Sciences in Zabrze, Medical University of Silesia, 41-902 Bytom, Poland

**Keywords:** non-alcoholic fatty liver disease, metabolic dysfunction-associated fatty liver disease, oxidative stress, antioxidant treatment, natural antioxidants, synthetic antioxidants, pro-oxidant–antioxidant balance, human studies, animal studies

## Abstract

Non-alcoholic fatty liver disease (NAFLD) is a global public health problem that causes liver-related morbidity and mortality. It is also an independent risk factor for non-communicable diseases. In 2020, a proposal was made to refer to it as “metabolic dysfunction-associated fatty liver disease (MAFLD)”, with concise diagnostic criteria. Given its widespread occurrence, its treatment is crucial. Increased levels of oxidative stress cause this disease. This review aims to evaluate various studies on antioxidant therapies for patients with MAFLD. A comprehensive search for relevant research was conducted on the PubMed, SCOPUS, and ScienceDirect databases, resulting in the identification of 87 studies that met the inclusion criteria. In total, 31.1% of human studies used natural antioxidants, 53.3% used synthetic antioxidants, and 15.5% used both natural and synthetic antioxidants. In human-based studies, natural antioxidants showed 100% efficacy in the treatment of MAFLD, while synthetic antioxidants showed effective results in only 91% of the investigations. In animal-based research, natural antioxidants were fully effective in the treatment of MAFLD, while synthetic antioxidants demonstrated effectiveness in only 87.8% of the evaluations. In conclusion, antioxidants in their natural form are more helpful for patients with MAFLD, and preserving the correct balance of pro-oxidants and antioxidants is a useful way to monitor antioxidant treatment.

## 1. Introduction

Non-alcoholic fatty liver disease (NAFLD) encompasses a range of liver conditions, from harmless non-alcoholic fatty liver (NAFL) to more severe non-alcoholic steatohepatitis (NASH) with or without fibrosis, NASH cirrhosis, and hepatocellular carcinoma (HCC) [1]. NAFLD is a significant public health concern, as it is a leading cause of liver-related morbidity and mortality globally, and it is also an independent risk factor for non-communicable diseases [2]. A combination of invasive and noninvasive tests is essential to diagnose NAFLD. The most comprehensive test for diagnosing and scoring fatty liver disease is a liver biopsy. The lesion at the most clinically benign end of the spectrum is fatty liver (hepatic steatosis). Both large (macro-) and microscopic (micro-) fat vesicles, mostly consisting of triglycerides, build up inside hepatocytes without significantly inducing scarring, liver cell death, or hepatic inflammation. The lesion at the other extreme end of the spectrum is known as cirrhosis. Hepatic steatosis frequently disappears by the time this level of architectural distortion manifests. Steatohepatitis is a type of liver damage that is characterized by hepatic steatosis and the development of restricted hepatic inflammation and hepatocyte death. Inflammatory infiltration is frequently evident in association with enlarged hepatocytes, which sometimes include Mallory’s hyalin. It consists of both mononuclear and polymorphonuclear leukocytes. These damage foci are primarily found in acinar zone 3 and are sometimes associated with bridging, perivenular, or perisinusoidal fibrosis [3]. The term proposed in 2020 to denote fatty liver disease associated with systemic metabolic dysregulation is “metabolic dysfunction-associated fatty liver disease (MAFLD).” The terminological transition from NAFLD to MAFLD was accompanied by a concise set of diagnostic criteria, facilitating convenient identification at the patient’s bedside for the broader medical community, including primary care providers [4].

Numerous interrelated mechanisms are involved in the pathophysiology of MAFLD. These processes include the infiltration of proinflammatory cells, which results in hepatic injury and ultimately leads to hepatic stellate cell (HSC) activation and fibrogenesis; lipotoxicity, which results from the accumulation of toxic lipid species; and insulin resistance (IR), which determines the metabolic syndrome. Although the proximal processes, such as inflammation, lipid excess, and lipotoxicity, have been extensively characterized, the downstream molecular mechanisms, including fibrogenesis, hepatocyte lipoapoptosis, and inflammatory processes, are not completely understood [5].

Liver diseases have a substantial impact on global health, with NAFLD being the most common worldwide, affecting 20–30% of the general population [6]. It affects 20–35% of adults, 15% of children, and up to 80% of individuals with obesity [7]. Cases increase markedly in patients with a history of type 2 diabetes mellitus (T2DM) and hyperlipidemia due to their connection to insulin resistance and metabolic dysfunction. However, NAFLD can affect individuals who have a normal weight and do not have metabolic problems, making up approximately 16% of cases [8]. Furthermore, NAFLD has led to an increase in death rates and liver transplants, particularly in the United States. Due to the fact that NAFLD is asymptomatic in its early stages, the actual burden of the disease can exceed the reported numbers [9]. The prevalence rates differ by region, ranging from 13.5% in Africa to 46% in America and an estimated 20–30% in Europe [2].

There is an opinion that a combination of several supportive treatments may be suitable for treatment. Due to the direct effect of body mass on NAFLD, weight loss treatment methods are used, the most important of which are changes in lifestyle through changes in eating habits and physical activity, as they reduce liver fat, and they should be used as the first line of treatment [10]. Over the past few decades, researchers have investigated several therapeutic agents that target different aspects of metabolic disorders, such as lipotoxicity, oxidative stress, mitochondrial dysfunction, and fibrosis, but some of these agents have been associated with disadvantages. However, the treatment used today includes antioxidants, such as vitamin E, and antidiabetic drugs, such as pioglitazone, and one of the treatments that has received a lot of attention in recent years is the targeting of intestinal bacteria [11,12].

Oxidative stress (OS) is characterized by an inconsistency between the generation of reactive species (RS) and antioxidant (AO) defenses [13]. A comprehensive description characterizes it as “an imbalance between oxidants and AOs, with oxidants having a greater advantage, resulting in a disturbance of redox signaling and regulation and/or molecular damage” [14]. OS is a crucial mechanism that contributes significantly to liver damage in MAFLD, playing a critical role in the transition from simple steatosis to NASH. Previous studies have obtained evidence showing that an increased production of reactive oxygen species (ROS) can trigger lipid peroxidation, resulting in inflammation and fibrogenesis via the activation of stellate cells [15]. Furthermore, ROS hinders the production of very-low-density lipoprotein (VLDL) by hepatocytes, leading to a buildup of fat in the liver. Additionally, ROS have the potential to induce hepatic insulin resistance and necroinflammation, as well as activate many intracellular pathways, which might ultimately result in hepatocyte apoptosis [16].

Different results have been obtained regarding the effect of antioxidants, which requires discussion. This systematic review aims to collect information on the impact of antioxidants in the treatment of MAFLD.

## 2. Materials and Methods

### 2.1. Literature Search

In this packccsystematic review, the recommendations stated in the Preferred Reporting Items for Systematic Reviews and Meta-Analyses (PRISMA) guidelines were followed. The systematic review protocol was registered in the PROSPERO database under the registration ID CRD42024534095, and a detailed exploration of the PubMed, SCOPUS, and ScienceDirect databases was performed. The primary sources of antioxidants are natural and dietary sources, as well as synthetic and medicinal supplements. Additionally, a search was performed using relevant keywords or title headings. Full details of the search strategy can be found in Table 1.

### 2.2. Study Selection

The inclusion and exclusion criteria for this review are described in Table 2. As an essential requirement for inclusion in the review, the research had to satisfy all established inclusion criteria. Publications were individually screened by two authors, KM and FF, to ensure compliance with the established inclusion criteria. Consequently, comprehensive reports were obtained for studies that indicated inconsistency or seemed to satisfy the inclusion criteria. Furthermore, disagreements were effectively resolved through an intensive evaluation process, resulting in the achievement of a consensus. On occasions when consensus could not be reached, the opinion of a third reviewer (SK) was sought.

## 3. Data Extraction and Quality Assessment

The reviewers autonomously extracted and documented data, including the author and publication year, population attributes such as sample size, antioxidant intervention (supplementation/dietary and medicinal forms), the duration of follow-up, post-intervention status, and quality control score determined by the two independent reviewers. The studies were evaluated using a study design and sampling method suitable for research. The sample size was sufficient, taking into account the prevalence of MAFLD. The evaluation of the results was performed using approved criteria. The outcomes were analyzed impartially, and the response rate was adequate. The statistics were reported with confidence intervals, and detailed descriptions of the study subjects were provided. Ultimately, the quality assessment determined that the selected studies met all the specified criteria and could be considered acceptable. 

## 4. Results

### 4.1. Search Results

**Figure 1:**
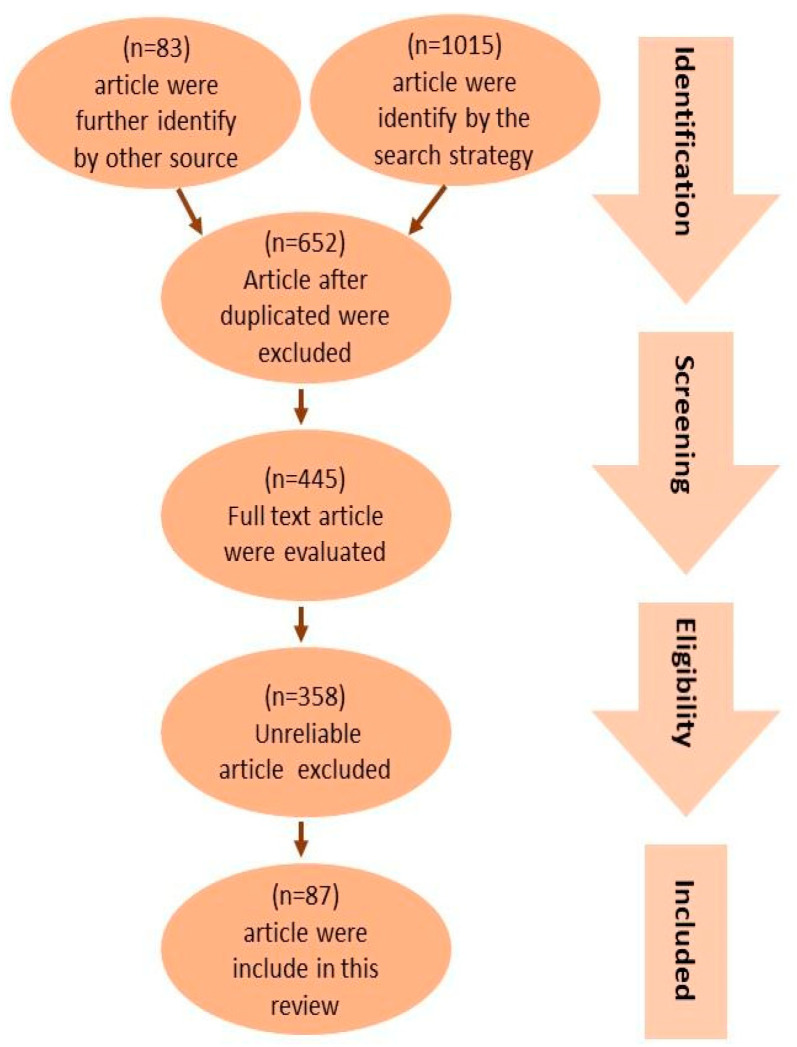
PRISMA flow diagram showing the study selection and identification. A total of 1015 publications were identified, with 535 articles in the human studies category and 480 articles in the animal studies category. By looking through the reference lists of pertinent reviews, an additional 83 publications were found, 38 of which were animal studies and 48 of which were human studies. After duplicates were eliminated, 653 articles were chosen for eligibility according to their title and abstract. Of these, 445 articles underwent full-text evaluations; 358 articles were omitted due to faulty data. As a result, in this review, 87 publications were included, with 45 being human studies and 42 being animal studies.

Figure 1 provides a concise overview of the procedure used to select pertinent studies. The search approach identified an overall number of 1015 articles. Furthermore, an additional 83 articles were found through an extensive review of the reference lists of pertinent reviews. Following the exclusion of duplicates, a total of 653 articles were selected based on their title and abstracts to determine their eligibility. A total of 445 articles were evaluated using their full texts, while 358 articles were removed due to factors such as untrustworthy study designs (including case reports, ethnographies, and observational designs), patient populations, interventions, outcome measurements, sample sizes smaller than 10, or the inaccessibility of the full text. As a result, a total of 87 articles were included in this review.

### 4.2. Study Characteristics

Of the 45 human studies, 14 (31.1%) used natural antioxidants, 24 (53.3%) used synthetic forms of antioxidants in dietary supplements and medications, and 7 used natural and synthetic antioxidants. Of the 24 studies that studied synthetic forms of antioxidants, 14 (58.8%) used dietary supplements, 4 (16.6%) used medications, and 6 (25%) used both dietary supplements and medications.

Among the human studies, 43 (95.5%) revealed a considerable effectiveness of antioxidant therapy, while 2 (4.4%) did not find significant differences from the placebo group. Of the 43 studies that had a significant effect, 14 examined natural antioxidants, 22 examined synthetic antioxidants, and 7 examined both natural and synthetic antioxidants. Furthermore, the two studies where no significant difference was seen examined synthetic antioxidants. Also, 30 studies examined both genders (66.6%), 8 studies (17.7) and 1 study (2.2%) included only males and females, respectively, and 6 studies did not mention the gender of cases. In addition, most of the studies, 38 of 45, have been designed as experimental, while there were 4 human studies, and 3 other studies used both experimental and human study design. The findings are summarized in Table 3.

Of the 42 animal studies, 10 (23.8%) used natural antioxidants, and 32 (76.1%) studies used synthetic forms of antioxidants in dietary supplements and medications. Of the 32 studies that studied synthetic forms of antioxidants, 7 (21.8%) used dietary supplements, and 25 (78.1%) studies used medications. 

Among the animal studies, a total of 38 investigations, representing 90.4% of the studies, indicated considerable effectiveness of antioxidant therapy. On the contrary, in four studies (9.5%), no significant changes were detected between the antioxidant therapy group and the placebo group. Of the 38 studies that indicated significant effectiveness, 10 examined natural antioxidants, and 28 studies examined synthetic antioxidants. All four studies where no significant difference was seen examined synthetic antioxidants. The findings are summarized in Table 4.

## 5. Discussion 

The present study was conducted as a systematic review to investigate the impact of antioxidant therapy on patients with MAFLD. This study investigated two types of antioxidants: natural antioxidants, found in fruits and vegetables, and synthetic antioxidants, such as dietary supplements or medications.

The analyzed studies indicate that natural antioxidants have a high level of efficacy in the treatment of MAFLD (100%). However, there is a scarcity of research using this method. The positive impact of synthetic antioxidants is not consistently observed, and only 91% of the studies demonstrate successful outcomes. On the basis of the available data, it may be inferred that natural antioxidants show greater efficacy than synthetic antioxidants in the treatment of MAFLD. Furthermore, fruits and vegetables are abundant in essential elements, as well as antioxidants. In contrast, previous studies have shown that the correct combination of minerals, such as sodium, potassium, selenium, magnesium, zinc, copper, and calcium, in conjunction with antioxidants, can improve the efficacy of antioxidants [104].

The impact of genetics on liver steatosis, inflammatory modifications, and fibrosis has been established by several studies. In genome-wide research, two genes have been associated with an increased risk of MAFLD: patatin-like phospholipase domain-containing 3 (PNPLA3) and trans-membrane 6 superfamily member 2 (TM6SF2) [9]. Lean MAFLD is a condition where individuals demonstrate a fatty liver but a normal body mass index (BMI). The main risk factors for this disease are visceral obesity, insulin resistance, high cholesterol, fructose intake, and specific genes. The triacylglycerol lipase enzyme, which is produced by the PNPLA3 gene, regulates lipid hydrolysis and assists in preserving balance between energy and its utilization in adipose tissue. Steatosis, inflammation, fibrosis, and hepatocellular carcinoma (HCC) can all be caused by a particular SNP in this gene. However, patients with MAFLD have a significantly decreased hepatic gene expression of TM6SF2, a gene essential for the generation of very-low-density lipoprotein (VLDL) [105]. An important gene, designated G-protein-coupled receptor 120 (GPR120), is present in hepatocytes, Kupffer cells, and adipocytes. It functions as a receptor for polyunsaturated fatty acids (PUFAs). Hepatocyte damage is the basis of abnormal liver function tests (LFTs) in individuals with the GPR120 270H mutation. Targeted by glitazone diabetes medications, PPARγ is frequently expressed in adipose tissue and influences both the arrival and departure of fatty acids to and from the liver, as well as the development of adipocytes. Liver steatosis is caused by these gene mutations [9].

Both in vitro and in vivo studies have shown that silybin or silibinin restores nicotinamide adenine dinucleotide+ (NAD+), a coenzyme essential for redox processes, by blocking poly (ADP-ribose) polymerase and triggering the SIRT1/AMP-activated protein kinase (AMPK) pathway. Reduced AMPK activity has been linked to the de novo lipogenesis process in MAFLD. Silybin’s anti-inflammatory action is achieved through the activity of SIRT2. Supplementing NAD+ with silybin has been found to be beneficial in preserving SIRT2 activity. Silybin has been shown to inhibit endoplasmic reticulum stress and the activation of the NLRP3 inflammasome in mice with fatty liver disease associated with metabolic dysfunction fed a high-fat diet [106].

Vitamin C has been reported to reduce mitochondrial ROS production, elevate levels of antioxidant enzymes such as superoxide dismutase and glutathione peroxidase, and enhance electron transport chain function in the liver. Vitamin C affects the balance of lipids and glucose and reduces visceral obesity and MAFLD by activating PPARα [9].

Multiple in vitro investigations on mouse and human adipocytes have shown that vitamin D has an anti-inflammatory impact by reducing the expression of chemokines and cytokines through the activation of p38 mitogen-activated protein (MAP) kinase and the NF-κB classical inflammatory pathway [9].

Vitamin E can boost antioxidant enzymes such as superoxide dismutase, catalase, and glutathione peroxidase; conversely, it reduces pro-oxidant contributions such as cellular myelocytomatosis (c-myc) and transforming growth factor-alpha (TGF-α), nitric oxide synthase, and NADPH. The antisteatotic activity of this substance is due to its capacity to decrease fatty acid uptake by hepatocytes via the downregulation of the hepatic cluster of differentiation 36 (CD36) protein, thereby limiting the amount of lipids available for peroxidation. Vitamin E reduces hepatic inflammation and fibrosis by downregulating the expression of pro-apoptotic BCL2 associated X (BAX), TGF-β, cyclooxygenase-2 (COX-2), and matrix metalloproteinase-2 (MMP-2) genes [1].

Plant flavonoids contain the naturally occurring flavonoid molecule quercetin. Quercetin’s antibacterial, anticancer, and antioxidant activities protect against free radicals, in addition to providing pharmacological advantages. One of the most abundant and substantial sources of quercetin is acacia rice. Numerous studies have demonstrated the effectiveness of quercetin, a flavonoid with potent antioxidant properties, in reducing lipid accumulation and the expression of SREBP1c and XBP-1 in adipocytes. A direct antilipogenic impact is produced by inhibiting the DNL pathway through its effects on the AMPK pathways [107].

Turmeric contains a polyphenolic compound known as curcumin, which has a number of medicinal effects, including anti-inflammatory, antiproliferative, antioxidant, and antiangiogenic effects. According to research, curcumin may prevent mice from developing MAFLD caused by high-fat and high-fructose diets. By regulating the LXR pathway, curcumin inhibits the expression of cytochrome P4503A (CYP3A) and cytochrome P4507A (CYP7A). Additionally, by inhibiting the FAS and Nrf2 pathways, it decreases the expression of CD36, SREBP1c, and the small heterodimer partner (SHP), which decreases hepatic steatosis. In an in vitro study, curcumin was found to reduce the development of liver fat by preventing citrate transport in the AMPK pathway, regulating the aberrant expression of SLC13A5/ACL. This prevents citrate from being carried or broken down. Curcumin inhibits the expression of SREBP1c and the suppressor of cytokine signaling 3 (SOCS-3), and it increases the phosphorylation of the hepatic activator of transcription 3 (STAT3), preventing liver steatosis. This assists in decreasing hepatic fat accumulation and regulating lipid metabolism [107]. A previous study demonstrated that, by enhancing the expression of the tight junction protein occludin 1, curcumin improved intestinal barrier function in MAFLD mice. The results of this study showed that it inhibited p65 nuclear translocation and NF-θB DNA-binding activity and that it decreased the expression of myeloid differentiation factor 88 (MyD88) in the liver, which, in turn, decreased hepatic steatosis [108]. MAFLD development is influenced by PPAR gene methylation. Curcumin significantly reduced methylation levels, elevated PPAR protein expression, and significantly reduced fat storage in MAFLD rats in other studies [109]. Curcumin protects LO2 cells from oleic acid-induced MAFLD by increasing the activity of certain proteins such as pAKT and P13K. Through Nrf2 signaling, it increases the absorption of glucose by liver cells and reduces NO and ROS levels. The initial phases of clinical trials have indicated that an excessive amount of 12 g/day of curcumin is safe for human intake. Because of its high metabolism and insufficient absorption, it has a low bioavailability. Currently, research is being conducted to increase the drug’s bioavailability and make it an unprecedented medication [107].

**Figure 2:**
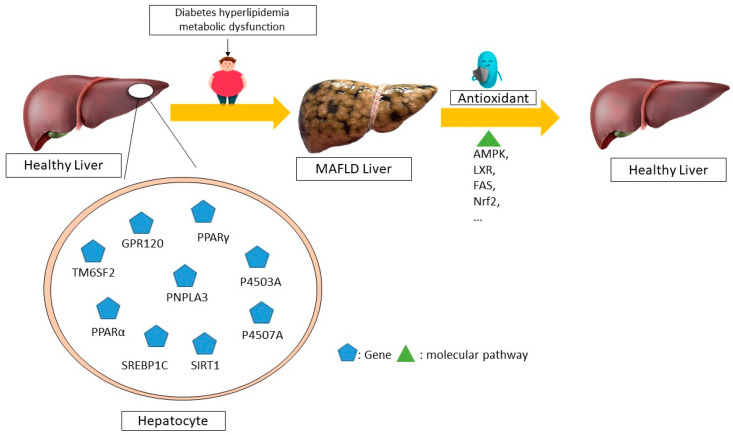
The molecular mechanisms underlying the regression of non-alcoholic fatty liver disease (NAFLD) mediated by antioxidants in humans. Different antioxidants can control the expression of different genes through distinct molecular pathways, leading to the recovery and regression of this disease, according to several human studies. For example, the disease is improved through the AMPK, LXR, FAS, and NRF2 pathways by altering the expression of the SIRT1, P4507A, P4503A, and SREBP1C genes, respectively. MAFLD—metabolic dysfunction-associated fatty liver disease; AMPK—AMP-activated protein kinase; LXR—liver X receptors; FAS—FS-7-associated surface; NRF2—nuclear factor erythroid 2-related factor 2; SIRT1—sirtuin 1; P4503A—cytochrome P450 3A; GPR120-G-protein-coupled receptor 120; TM6SF2-trans-membrane 6 superfamily member 2; PNPLA3-patatin-like phospholipase domain-containing 3; PPARα-Peroxisome proliferator-activated receptor α; P4507A—cytochrome P450 7A; SREBP1C—sterol regulatory element binding protein 1c.

Resveratrol is a phenolic molecule that belongs to the stilbene family of phenols. It has a structure of C6-C2-C6 and is commonly found in plants such as cassia seeds, grape skins, and white tea. The substance comes primarily from the rhizome extract of Polygonum multiflorum. Resveratrol exhibits potent antioxidant, anticancer, and anti-inflammatory properties. Research has shown that resveratrol stimulates the sirtuin pathway (STRT1) for the treatment of MAFLD. Resveratrol reduces fat storage by activating the STRT1-FOXO1 pathway, which prevents SREBPE-1c acetylation and decreases metabolic abnormalities. A reduction in STRT1 levels in hepatocytes can lead to liver inflammation [110]. The stimulation of 3T3-L1 cells with TNF-ɑ leads to an increase in cytokine mRNA expression through the siRNA-mediated activation of SIRT1 [107]. Meanwhile, elevated SIRT1 levels suppress the generation of pro-inflammatory cytokines such as NF-ĸB and TNF-ɑ, thus protecting the liver’s metabolism from the harm caused by a high-fat diet. Research has shown that resveratrol can decrease apoptosis, mitochondrial dysfunction, and reactive oxygen species (ROS) production in OA-induced L02 cells. It can treat metabolic dysfunction-associated fatty liver disease (MAFLD) by decreasing caspase-3 and p53 and increasing B-cell lymphoma 2 (Bcl-2) levels, which helps to reduce liver fat accumulation [111]. The molecular mechanisms of non-alcoholic fatty liver disease (NAFLD) mediated by antioxidants in humans is summarized in Figure 2.

Furthermore, in 4.4% of the studies analyzed, there was no statistically significant impact on patient improvement when antioxidants were used. Furthermore, the reviewed studies also indicated that the efficacy of antioxidant therapy was not significantly influenced by the dosage or duration of treatment.

In the present systematic review, a study of animal research was also conducted. The reviewed animal studies showed that natural antioxidants are thoroughly effective in treating MAFLD (100%), but few studies used this method. This beneficial effect was not consistently seen with synthetic antioxidants, and only 87.8% of the studies showed effective results. On the basis of these data, it can be assumed that antioxidants may be more effective in their natural form than in a synthetic form in treating MAFLD.

Feeding a high-fat diet (HFD) to mice leads to the accumulation and fibrosis of liver collagen. Ascorbic acid supplementation decreases collagen levels and the mRNA expression of TGF-β and collagen. It also inhibits hepatocyte apoptosis and liver injury by increasing Bcl-2 protein levels and decreasing caspase 8. It effectively reduces liver inflammation in obese mice by suppressing the expression of inflammatory genes, including TNFα and MCP-1, in hepatocytes [112].

By controlling the proteins involved in lipid homeostasis, in which CES1 activation plays a crucial role, vitamin E in mice reduces the effects of a high-fat diet on liver lipid accumulation and glucose homeostasis. The biochemical mechanism responsible for the vitamin E-mediated overexpression of CES1 is, at least partially, the activation of nuclear Nrf2 [113].

Quercetin may help HFD-fed mice overcome MAFLD. An HFD causes fat peroxidation, ferroptosis, and fat accumulation; therapy clearly reverses these effects. For example, quercetin inhibits ferroptosis by increasing the expression of anti-glutathione peroxidase four (GPX4) and decreasing the expression of anticyclooxygenase 2 (COX2) and the long-chain family member acyl-coenzyme A synthase four (ACSL4) [114].

**Figure 3:**
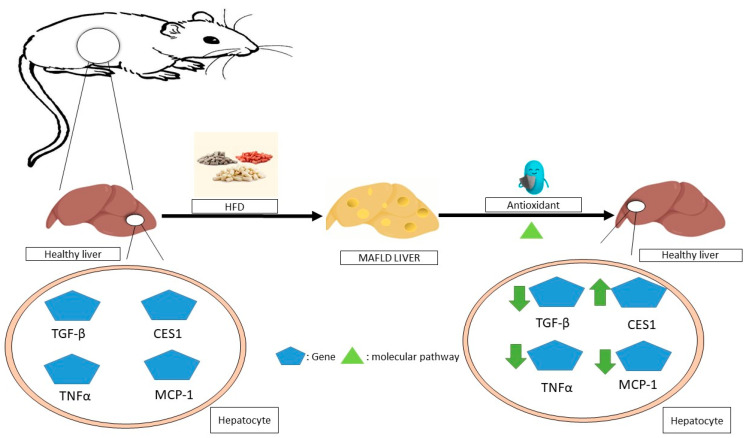
The molecular mechanisms underlying the regression of non-alcoholic fatty liver disease (NAFLD) mediated by antioxidants in animals. In animal studies, antioxidants have been shown to improve this disease by affecting the expression of various genes in hepatocytes through molecular pathways, for example, by reducing the expression of inflammatory genes such as TNFα and MCP-1, as well as reducing the expression of TGF-βmRNA or the overexpression of CES1. MAFLD—metabolic dysfunction-associated fatty liver disease; TNF-α—tumor necrosis factor alpha; MCP-1—monocyte chemoattractant protein-1; TGF-β—transforming growth factor beta; CES1—carboxylesterase 1.

The administration of resveratrol to obese mice improves liver steatosis by improving glycolipid metabolic parameters, liver histology, inflammation, and lipid content. It could potentially have further positive effects by increasing T-SOD and GPX activities, inhibiting TNF-α production, suppressing TLR4 and CD36 expression [115], and improving steatohepatitis through the inhibition of the NF-jB inflammation pathway by further activating the AMPKa-SIRT1 pathway [116]. The molecular mechanisms of non-alcoholic fatty liver disease (NAFLD) mediated by antioxidants in animals is summarized in Figure 3.

In general, research examining the impact of antioxidants on the improvement of the condition of patients with MAFLD reveals contradictory results. On the contrary, the observed contradiction related to antioxidant properties in MAFLD therapy could also be attributed to the oxidative impact of antioxidants. Research has shown that, when the concentration of antioxidants in the human body is above the necessary threshold, oxidative effects are increased. In addition, an excess of antioxidants can contribute to elevated levels of oxidative stress [117]. Therefore, despite the positive therapeutic perspective of antioxidant supplements, it is essential to exercise caution and avoid their indiscriminate and/or excessive administration. The cellular intrinsic antioxidant system provides protection against damage caused by oxidants. Therefore, an assessment of the pro-oxidant–antioxidant balance can serve as an effective strategy for the management of antioxidant therapy. Measurement of oxidant and antioxidant capacity has been shown to facilitate the understanding of their balance. As a result, the administration of antioxidants may provide greater efficacy in patients when considering the balance or ratio between pro-oxidants and antioxidants [110,118].

The efficacy of antioxidants as an alternative therapy for MAFLD may be limited due to its multifactorial nature. The use of supplementary treatments based on the patient’s underlying disease, in conjunction with a suitable dose of antioxidants, appears to result in greater efficacy. The concept of combination therapy is interesting because it allows for the simultaneous targeting of multiple pathologic contributors to the disease. In the context of hepatic fibrosis, combination drugs can also be used to address the same target. In this context, these medications may have additive or synergistic effects on the target. Furthermore, the inclusion of a drug can potentially lead to a reduction in the dosage of other medication, thereby improving safety. It should be noted that the inclusion of a drug may reduce the negative consequences associated with an otherwise efficacious drug [119,120]. Furthermore, many fruits and vegetables, such as oranges, contain potassium and calcium, which have the potential to effectively manage blood pressure. On the basis of the evidence mentioned above, it can be inferred that the natural antioxidants present in fruits and vegetables may demonstrate greater effectiveness [104]. It is possible that medicinal supplements contain a combination of ingredients consisting of natural antioxidants that are effective. This study also highlights the absence of other comparable studies investigating the efficacy of antioxidants in preventing MAFLD in at-risk groups.

## 6. Current Limitations of Knowledge on the Effectiveness of Antioxidants in Treating MAFLD and Their Prospects

Although some evidence suggests the possible advantages of antioxidants for MAFLD, there are still certain issues that need to be clarified. First, because the vast majority of studies had an average duration of less than six months or 1 year, there has been an absence of substantial and outstanding scientific evidence indicating the long-term advantages of MAFLD antioxidant treatment. Second, it is currently questionable whether the general population and specific populations, such as men and women, the elderly, those with diabetes mellitus, children, and adolescents, should be prescribed antioxidant treatment. Third, how does long-term use of this antioxidant treatment affect people’s health? The essential concern is to assess who will benefit the most from an antioxidant treatment for MAFLD based on their personal characteristics, especially those with overlapping health conditions. Fourth, it is challenging to determine consistent findings because the included studies varied according to the characteristics of their study structure, patient demographics, antioxidant types, dosages, and treatment durations. Fifth, confounding variables can affect the results and make it challenging to differentiate between the impact of antioxidants. These variables include differences in the initial characteristics of the study participants, lifestyle factors, and concurrent treatments. Sixth, data analysis may be limited by the variations in the methods used by studies to assess and present outcomes associated with antioxidant levels, metabolic parameters, and liver function.

## 7. Conclusions

Antioxidants have been indicated to have similar effects on humans and animals. These effects include regulating the SIRT1/AMPK, NFKB, and LXR pathways; decreasing the expression of proapoptotic genes such as BAX, inflammatory genes such as TNF-ɑ, as well as TGF-β and COX2 genes; inhibiting the FAS and NrF2; and activating PPARα. Although natural antioxidants may be beneficial in treating MAFLD patients, combination therapy has been demonstrated to be more effective. However, more research is required to completely comprehend the effect of natural antioxidants. To improve the efficacy of MAFLD treatment, it is essential to take into account two crucial criteria. An effective approach to controlling treatment with antioxidants involves evaluating the appropriate balance between pro-oxidants and antioxidants. In essence, the administration of antioxidants at a suitable dose improves their efficacy, indicating the need for the development of customized therapeutic approaches. Furthermore, it is highly recommended to use the ideal dose of antioxidants in conjunction with other medications, such as antihypertensives, hypoglycemic agents, antidiabetic agents, and other cholesterol-balancing therapies, to effectively treat the underlying condition in patients with MAFLD.

## Figures and Tables

**Table 1:** Full details of the search strategy terms.

Terms ^1^	Search Strategy Terms
Term 1	“Antioxidants” OR “Antioxidant treatments” “Antioxidant therapy”
Term 2	“Antioxidant drugs” OR “vitamin C” OR “Ascorbic acid” OR “Vitamin A” OR “Vitamin D” OR “Vitamin E” OR “Resveratrol” OR “Tocopherol” OR “Pentoxifylline” OR “Silymarin” OR “Green Tea” OR “Chocolate” OR “Garlic” OR “Ginger” OR “Red Wine”
Term 3	Consumption OR “Dietary intake” OR Supplement OR Supplementation OR “Nutritional supplement” OR “high-fat diet” OR “chow”
Term 4	“Non-alcoholic fatty liver disease” OR “Metabolic dysfunction-associated fatty liver disease” OR “NAFLD” OR “MAFLD”
Term 5	“Clinical Trials” OR “Animal Study” OR “Mice” OR “Rat” OR “Rabbit” OR “Guinea pig”

^1^ Terms 1, 2, 3, and 4 were joined with “AND”.

**Table 2:** Inclusion and exclusion criteria.

Inclusion Criteria	Exclusion Criteria
Human studies	Human studies
Studies that include men (males) and women (females)	
Studies that are randomized controlled trials	Studies that include participants at risk of MAFLD, including otherwise healthy smokers and hypertensive or diabetic patients
Studies with a sample size of 10 participants	Studies that administer mixed nutrient supplementation where no group receives any specific antioxidant supplement alone
Studies that include participants who either are healthy or have established MAFLD	Studies that incorporate dual treatments such as exercise and supplementation Studies in which there are no data relating to the dose of natural antioxidants Studies in which there is no control group
Studies that orally administer a single antioxidant intervention through supplementation or dietary or drug interventions	
Animal studies	Animal studies
Studies that include males and females	
Studies that include participants in whom the diet induced MAFLD	Studies that apply other treatments along with antioxidants
Studies that provide an oral or injectable antioxidant intervention	Studies that administer mixed nutrient supplementation where no group receives any specific antioxidant supplement alone
Studies that administer a single antioxidant or a combination of antioxidants through supplementation or dietary intervention	

**Table 3:** The summarized results of human studies that fulfilled the inclusion criteria.

Study	Year	Type of Study	Sample Size	Age	Gender	Intervention	Form of Intervention (Natural or Synthetic)	Dose/Day	Duration	Control	Status after Intervention
Mansour-Ghanaei et al. [17]	2019	Experimental	228	Mean age: 50	Not mentioned	Curcumin	Natural: curcumin	80 to 3000 mg/day	Ranged between 8 and 12 weeks	There were no controls in this study	Increased dosages of curcumin/turmeric may be beneficial for NAFLD.
Mosca et al. [18]	2020	Experimental	80	Adolescents (age range: 4–16 years)	Not mentioned	Group 1: vitamin E and hydroxytyrosol Group 2: placebo	Synthetic: vitamin E	Not mentioned	4 months	Placebo	Hydroxytyrosol and vitamin E significantly decreased IL-6, which, in turn, decreased systemic inflammation. The combination increased the expression of IL-10; this can hinder the release of cytokines, which increase inflammation.
Khachidze et al. [19]	2019	Both human and experimental	107	Not Mentioned	Not mentioned	Group 1: vitamin E Group 2: lifestyle modification Group 3: ursodeoxycholic acid (UDCA)	Synthetic: vitamin E	Group 1: 400 IU once daily Group 2: __ Group 3: 15 mg/kg once daily	12 months	Placebo	Combining vitamins E and C is a safe, affordable, and effective therapeutic option for patients with NASH. This may help minimize oxidative stress-related damage and prevent the progression of cirrhosis.
Anushiravani et al. [20]	2019	Experimental	150	Aged between 18 and 65 years	Both genders	Group 1: vitamin E Group 2: placebo Group 3: metformin Group 4: silymarin Group 5: pioglitazone	Synthetic: vitamin E and metformin and silymarin and pioglitazone	Group 1: vitamin E 400 IU once daily Group 2: placebo Group 3: metformin 500 mg once daily Group 4: silymarin 140 mg once daily Group 5: pioglitazone 15 mg once daily	3 months	Placebo	In NAFLD patients, vitamin E, silymarin, and pioglitazone were shown to improve liver aminotransferases.
Barchetta et al. [21]	2011	Experimental	55	57.4 ± 10.7	Females = 20 Males = 35	Vitamin D (cholecalciferol)	Synthetic: vitamin D	2000 IU/day	24 weeks	Not mentioned	Clinical studies did not demonstrate that vitamin D treatment has a beneficial effect on liver impairment markers in MAFLD patients.
Sanyal et al. [22]	2010	Experimental	*n* = 247 Group 1 (pioglitazone): 80 Group 2 (vitamin E): 84 Group 3 (placebo): 83	46.3	Females: 60% Males: 40%	Group 1: pioglitazone Group 2: vitamin E	Synthetic: vitamin E and pioglitazone	Group 1: 30 mg once daily Group 2: 800 IU once daily	2.5 years	Group 3: placebo	Vitamin E was more effective than placebo in treating non-alcoholic steatohepatitis in persons without diabetes. Pioglitazone did not provide any advantages over placebo in the main outcome, although it did show substantial benefits for some secondary outcomes.
Foster et et al. [23]	2011	Experimental	*n* = 455 Group 1 (atorvastatin, vitamin C, and vitamin E): 229 Group 2 (placebo): 226	59	Females: 29.1% Males: 70.9%	Atorvastatin, vitamin C, and vitamin E	Synthetic: vitamin C, vitamin E, and atorvastatin	Atorvastatin 20 mg, vitamin C 1 g, and vitamin E 1000 IU once daily	4 years	Placebo	The combination of atorvastatin 20 mg with vitamins C and E was found to effectively reduce the likelihood of hepatic steatosis by 71% in healthy adults with non-alcoholic fatty liver disease (NAFLD) at baseline.
Hoofnagle et al. [24]	2013	Experimental	139	45.5	Females: 57.5% Males: 42.5%	Group 1: pioglitazone 30 mg and vitamin E placebo Group 2: vitamin E 80 once daily and pioglitazone placebo	Synthetic: vitamin E and pioglitazone	Group 1: pioglitazone 30 mg and vitamin E placebo Group 2: vitamin E once daily and pioglitazone placebo	2.5 years	Placebo	Patients with NASH showed a reduction in ALT levels, as well as an improvement in histological activity.
Polyzos et al. [25]	2017	Experimental	*n* = 31 Group 1 (vitamin E alone): 17 Group 2 (spironolactone plus vitamin E): 12	Study included individuals ≥18 years)	Females: 74.2% Males: 25.8%	Group 1: vitamin E Group 2: spironolactone plus vitamin E	Synthetic: vitamin E and spironolactone	Group 1: vitamin E 400 IU twice a day Group 2: spironolactone 25 mg once a day plus vitamin E 400 IU twice a day	52 months		The combination of low-dose spironolactone and vitamin E resulted in a considerable reduction in the NAFLD liver fat score.
Pervez et al. [26]	2018	Experimental	*n* = 64 Group 1 (δ-tocotrienol): 31 Group 2 (placebo): 33	44.3	Females: 54.7% Males: 45.3%	δ-tocotrienol	Synthetic: δ-tocotrienol	Group 1: δ-tocotrienol 300 mg twice daily	3 months	Placebo	The administration of δ-tocotrienol substantially increased AST activity and reduced inflammatory and oxidative stress markers in individuals diagnosed with NAFLD.
Bril et al. [27]	2019		*n* = 105 Group 1 (vitamin E plus placebo): 36 Group 2 (vitamin E plus pioglitazone): 37 Group 3 (both placebo): 32	59	Female: 11.4% Males: 88.6%	Group 1: vitamin E plus placebo Group 2: vitamin E plus pioglitazone	Synthetic: vitamin E and pioglitazone	Group 1: vitamin E 400 IU twice a day plus placebo Group 2: vitamin E 400 IU twice a day plus pioglitazone 30 mg/day increased after 2 months to 45 mg/day	18 months	Placebo	Combination therapy provided better outcomes than a placebo in enhancing liver histology in patients with NASH and T2DM. The administration of vitamin E alone did not have any significant effect on the primary histological outcome.
Asghari et al. [28]	2018	Both human and experimental	60	39.53	Males: 58.33%	Pure trans- resveratrol	Synthetic: capsules, pure trans-resveratrol	600 mg\day	84 days	Placebo	A calorie-restricted (CR) diet resulting in moderate weight loss had beneficial effects on NAFLD. Furthermore, weight loss induced by resveratrol supplementation also had positive effects. However, it should be noted that these interventions did not replicate all the benefits of a CR diet.
Chachay et al. [29]	2014	Experimental	20	48.15	Males: 100%	Resveratrol	Synthetic: capsules	3000 mg\day	56 days	Placebo	The administration of resveratrol did not result in significant improvements in any of the characteristics of NAFLD when compared to a placebo. However, it did result in an increase in hepatic stress, demonstrated by an observed increase in the levels of liver enzymes.
Chen et al. [30]	2015	Experimental	60	44.30	Males: 70%	Resveratrol	Synthetic: capsules (from natural products)	600 mg\day	90 days	Placebo	Resveratrol reduced levels of ASPAT, ALAT, LDL-cholesterol, total cholesterol, TNF-α, cytokeratin fragment 18, and fibroblast growth factor 21, as well as the IR index, in patients with NAFLD. This suggests that resveratrol supplementation may be beneficial for individuals with NAFLD.
Heebøll et al. [31]	2016	Experimental	28	43.2	Males: 65.38	Pure trans-resveratrol	Synthetic: capsules, pure trans-resveratrol	1500 mg\day	180 days	Placebo	The administration of resveratrol did not consistently improve the clinical or histological symptoms of NAFLD. However, there may have been a slight improvement in liver function tests and a reduction in the accumulation of liver fat.
Pezeshki et al. [32]	2016	Experimental	71	35	Both genders	Green tea	Synthetic: green tea extract (GTE) tablets	500 mg/day	84 days	Placebo (cellulose)	In NAFLD patients, GTE supplementation decreased liver enzyme levels. It is possible to suggest that administering GTE to NAFLD patients can help them achieve better blood liver enzyme levels.
Hussain et al. [33]	2017	Experimental	80	26.5	Both genders	Green tea	Synthetic: capsule (GTE)	1000 mg/day	84 days	Placebo (cellulose)	The metabolic, chemical, inflammatory, and radiological characteristics of patients with NAFLD and dyslipidemia but without diabetes significantly improved as a result of GTE therapy.
Tabatabaee et al. [34]	2017	Experimental	45	40	Both genders	Green tea	Synthetic: green tea tablets	550 mg/day	90 days	Placebo	The two groups mainly differed in anthropometrics, liver enzyme levels, and metabolic markers; however, the results of the routine therapies that both groups received may have concealed some of the differences.
Sakata et al. [35]	2013	Experimental	17	55.9	Both genders	Green tea	Synthetic: green Tea	Group 1: 1080 mg/ 700 mL catechins Group 2: mg/700 mL catechins	84 days	Placebo (green tea-flavored beverage)	The consumption of a high-catechin tea reduced blood biochemistry, liver inflammation, and liver fat in addition to an oxidative stress marker.
Fukuzawa et al. [36]	2014	Experimental	38	50	Both genders	Green tea	Synthetic: green tea	Dietary and exercise therapy + 600 mg/ day catechins	180 days	Dietary and exercise therapy	Green tea catechins have the capability to inhibit NASH by efficiently improving NASH-related parameters such as insulin resistance recovery and anti-inflammatory activities.
Rezaei et al. [37]	2019	Experimental	66	Age ≥ 18 years	Not mentioned	Olive oil	Natural: olive oil	20 g per day	12 weeks	Sunflower oil	In NAFLD patients, olive oil decreased body fat percentage and attenuated fatty liver grade; it had no effect on liver enzymes or cardiometabolic risk factors.
Sofi et al. [38]	2010	Both human and experimental	11	Age > 18 years	Males: four Females: two	Olive oil with n-3 PUFA	Synthetic: olive oil with n-3 PUFA	6.5 mL per day	12 months	Dietary recommendations and a similar package of olive oil not enriched with n-3 PUFA	Patients with NAFLD may significantly increase their adiponectin levels and reduce their circulating triglycerides and liver enzymes via the long-term use of olive oil enriched with n-3 PUFA.
Shidfar et al. [39]	2018	Experimental	50	45.91 ± 9.61	19 women and 31 men	Olive oil	Natural: olive oil	A hypocaloric diet enriched with olive oil (20% of the total energy intake)	12 weeks	A hypocaloric diet with normal fat	A normal fat percentage of 30% in a diet containing olive oil (with the consumption of 20% of total calories from virgin olive oil) combined with a slight decrease in weight (about 5%) supported the intended effects of weight loss in increasing the levels of the enzymes ALT and AST.
Nigam et al. [40]	2014	Experimental	93	Age from 20 to 50 years	Males: 100%	Olive oil, canola oil, and soybean	Natural: olive oil, canola oil, and soybean	Cooking medium (not exceeding 20 g/day)	6 months	Safflower oil	The severity of fatty liver and liver span in NAFLD significantly reduced when olive and canola oils—which are high in MUFAs and have a balanced n-6/n-3 PUFA ratio—were used as a cooking medium. Insulin resistance and dyslipidemia both improved along with the fatty liver.
Cueto-Galán et al. [41]	2017	Human study	276 participants, 57% NAFLD	67	Females: 66% Males: 34%	Mediterranean diet (MD) with extra virgin olive oil and MD with nuts	Natural: MD with extra virgin olive oil and MD with nuts	MD with extra virgin olive oil (1 l/week), MD with nuts (30 g/day)	6-year follow-up	Low-fat diet	A Mediterranean diet could be used as a dietary intervention to prevent or treat NAFL by preventing or reducing the disease’s natural course.
Kontogianni et al. [42]	2014	Human study	73	18–65 years old	Both genders	MD via MedDietScore	Natural: MD via MedDietScore	_	12 months	Stable dietary and exercise habits	Although increased Mediterranean diet adherence was associated with less severe liver disease and a reduced level of insulin resistance in NAFLD patients, it was not associated with a decreased likelihood of developing the disease.
Ryan et al. [43]	2013	Human study	12	Not Mentioned	6 females/6 males	MD	Natural: MD	_	6-week wash-out period in between	Control diet (low-fat–high-carbohydrate diet)	Compared to current dietary recommendations, MD improved insulin sensitivity and decreased liver steatosis in an insulin-resistant NAFLD population, even in the absence of weight loss.
Trovato et al. [44]	2015	Human study	90	50.13 ±13.68	Females: 46 Males: 44	MD diet intervention	Natural: MD diet intervention	_	6 months	_	Maintaining the Mediterranean diet is a strong indicator of alterations in the liver’s fat composition in overweight NAFLD patients. The diet has a beneficial, progressive effect that occurs on its own without requiring other lifestyle modifications.
Zhang et al. [45]	2015	Experimental	74	Age from 25 to 65	Both genders	Anthocyanin\red orange juice and raw red raspberry (equivalent dose from dietary source (according to Phenol Explorer))	Synthetic: anthocyanin Natural: red orange juice and raw red raspberry	Anthocyanin 320 mg, once a day\3.17 mg/100 mL red orange juice, 72.47 mg/100 g of raw red raspberry	12 weeks	Placebo	Dietary supplements containing anthocyanins may reduce the symptoms of NAFLD. Furthermore, these supplements are an inexpensive, effective treatment that can improve general health by decreasing the risk of T2DM, cardiovascular disease, and chronic liver disease at the same time.
Suda et al. [46]	2008	Experimental	48	30–60 years	Males: 100%	Purple sweet potato beverage (anthocyanins)\red orange juice and raw red raspberry (equivalent dose of dietary source (according to Phenol Explorer))	Synthetic: purple sweet potato beverage (anthocyanins) Natural: red orange juice and raw red raspberry	125 mL/bottle, 2 bottles per day\3.17 mg/100 mL red orange juice, 72.47 mg/100 g of raw red raspberry	8 weeks	Placebo	Hepatic biomarker serum levels were considerably reduced after consuming the purple sweet potato beverage.
Faghihzadeh et al. [47]	2014	Experimental	50	44.04	Males: 18 (72%)	Resveratrol\red wine and black grapes (equivalent dose from the dietary source (according to Phenol Explorer))	Synthetic: resveratrol Natural: red wine; black grapes	Resveratrol 500 mg, once a day\0.15 mg/100 mL red wine; 0.18 mg/100 g black grapes	12 weeks	Placebo	For the treatment of NAFLD, resveratrol supplementation is favorable to lifestyle modifications alone. The body’s hepatocellular apoptosis and the attenuation of inflammatory indicators are at least substantially accountable for this.
Stiuso et al. [48]	2014	Experimental	30	_	Not mentioned	Silymarin (Realsil, silybin, phosphatidylcholine, ɑ-tocopherol\dried milk thistle fruit (112) (equivalent dose from dietary source (according to Phenol Explorer))	Synthetic: silymarin Natural: dried milk thistle fruit	Realsil, 94 mg silybin, 194 mg phosphatidylcholine, 30 mg ɑ-tocopherol, twice a day\2.68% *w*/*w* of dried milk thistle fruit (112)	12 months	_	Realsil may be effective in improving a patient’s metabolic asset if they have moderate NASH. In conclusion, this research indicates that the lipidomic profile of these RA patients changed in a distinctive way as a result of their therapy. This is probably because every patient has a different metabolic response, and they should be further categorized depending on other metabolic factors such as age, sex, AST, ALT, and GGT levels.
Loguercio et al. [49]	2012	Experimental	179	Age 18–65	Not mentioned	Silymarin (Realsil, silybin, phosphatidylcholine, ɑ-tocopherol\dried milk thistle fruit (equivalent dose from dietary source (according to Phenol Explorer))	Synthetic: silymarin Natural: dried milk thistle fruit	Realsil, 94 mg silybin, 194 mg phosphatidylcholine, 30 mg ɑ-tocopherol, twice a day\2.68% *w*/*w* of dried milk thistle fruit	12 months	Placebo	Realsil-treated NAFLD patients showed improvements in insulin resistance, liver histology, γGT levels, and transaminase levels without increasing weight.
Solhi et al. [50]	2014	Experimental	64	Patients in case group: 43.6 ± 8.3 Patients in control groups: 39.4 ± 10.5	Both genders	Silymarin\ dried milk thistle fruit (equivalent dose from dietary source (according to Phenol Explorer))	Synthetic: silymarin Natural: dried milk thistle fruit	Silymarin 70 mg, three times a day\2.68% *w*/*w* of dried milk thistle fruit	8 weeks	Placebo	For the treatment of NASH, silymarin is a beneficial herbal medication. Patients who consumed it showed a more noticeable decrease in their hepatic enzyme levels.
Soleimani et al. [51]	2020	Experimental	110	Treatment: 45.6 ± 11.3 Control: 42.9 ±1 2.21	Both genders	Garlic powder tablets	Synthetic: garlic powder tablets	400 mg garlic powder tablets	15 weeks	Placebo	Supplementation with garlic could assist NAFLD patients in decreasing body fat mass. Garlic may consequently decrease liver fat levels and prevent or impede the development of NAFLD.
Sangouni et al. [52]	2020	Experimental	90	Treatment: 45.2 ± 12.4 Control: 44.2 ± 11.1	Both genders	Garlic tablets daily	Synthetic: garlic tablets daily	Four garlic tablets daily (each tablet contained 400 mg garlic powder)	12 weeks	Placebo	This clinical experiment demonstrated that supplementing with garlic powder improves liver enzymes, lipid profile, and hepatic steatosis as a dyslipidemia indicator. Therefore, implementing garlic into a patient’s adjusted diet seems reasonable and is an essential aspect of the treatment strategy for NAFLD.
Kim et al. [53]	2017	Experimental	75	Treatment: 54.5 ± 12.2 Control: 54.2 ± 9.3	Both genders	Garlic	Natural: garlic sachets	Two sachets of garlic/per day	12 weeks	Placebo	For individuals without underlying hepatic disease, fermented garlic extracts (FGEs) might serve as a reliable and beneficial treatment for fatigue and reduce hepatic dysfunction caused by oxidative stress and inflammation.
Zhang et al. [54]	2019	Experimental	24,106	Males: 41.0 ± 12.2 Females: 40.3 ± 11.6	Both genders	Raw garlic	Natural: raw garlic	Garlic (≥7 t/week vs. <1 t/week) Garlic (4–6 t/week vs. <1 t/week) Garlic (1–3 t/week vs. <1 t/week) Garlic ≥7 t/week vs. <1 t/week) Garlic (4–6 t/week vs. <1 t/week) Garlic (1–3 t/week vs. <1 t/week)			Consuming raw garlic consistently is inversely associated with NAFLD.
Emamat et al. [55]	2020	Experimental	999	Cases: 42.3 ± 11.9 Control: 43.5 ± 14.5	Both genders	Dietary allium vegetable intakes	Natural: dietary allium vegetable intake	7.17 (2.52–15.21) g/day			Higher allium vegetable consumption was associated with a decreased incidence of NAFLD.
Loffredo et al. [56]	2017	Experimental	57 (3 groups: NASH (*n* = 19) FLD (*n* = 19) Controls (*n* = 19)	NASH (46 ± 11) FLD (46 ± 6) Controls (47 ± 8)	Both genders (males/females: 11/8 per group)	NASH: dark chocolate, cocoa >85% and milk chocolate, cocoa <35%	Natural: dark chocolate and milk chocolate	40 g/day dark chocolate and 40 g milk chocolate	2 weeks		By comparing patients with NASH with individuals with fatty liver disease (FLD) and healthy controls, it was found that the former showed decreased flow-mediated dilation (FMD) and higher NOX2 activation and oxidative stress. In NASH patients, dark chocolate but not milk chocolate can improve endothelial function.
Rafie et al. [57]	2020	Experimental	Ginger group: 23 Control group: 23	Ginger group: 50.04 ± 10.26 Control group: 47.95 ± 9.24	Both genders	Ginger powder	Natural: ginger powder	1500 mg ginger powder daily	12 weeks	Placebo	Individuals with NAFLD responded more effectively to dietary recommendations and lifestyle modifications.
Daneshi-Maskooni et al. [58]	2019	Experimental	Ginger group: 43 Control group: 44	Ginger group: 45.5 ± 8. 9 Control group: 45.0 ± 7.7	Both genders	Capsules s (green cardamom (GC) from ginger family)	Synthetic: capsules (GC from ginger family)	Two 500 mg capsules (GC from ginger family) three times per day	3 months	Placebo	It is likely that, by increasing serum Sirt1 and irisin levels, GC supplementation reduced fatty liver grade, blood glucose indices, and lipid profiles in NAFLD patients with overweight or obesity.
Rahimlou et al. [59]	2016	Experimental	Ginger group: 23 Control group: 21	Ginger group: 45.45 ± 2.31 Control group: 45 ± 2.14	Both genders	Ginger capsule	Synthetic: ginger capsule	Two 500 mg ginger capsules twice daily (2 g daily)	12 weeks	Placebo	Supplementation with ginger was shown to improve certain NAFLD features, as well as decrease hs-CRP, TNF-α, ALT, GGT, hepatic steatosis, and HOMA-IR.
Anty, R et al. [60]	2012	Experimental	195	39 ± 12.3 years	Both genders	Coffee-containing beverages: espresso; double espresso; filtered (regular); made with Italian coffee machine; decaffeinated coffee	Natural: coffee-containing beverages: espresso; double espresso; filtered (regular); made with Italian coffee machine; decaffeinated coffee	-	20 months	Placebo	Regarding individuals who routinely drank coffee, the quantity of caffeine consumed and the severity of fibrosis were negatively correlated. Consuming coffee on a regular basis is one independent way to prevent fibrosis. There is no evidence linking the consumption of espresso coffee to the onset of fibrosis.
Zelber-Sagi, S et al. [61]	2014	Experimental	347	50.86 ± 10.35 years	Both genders	All types of caffeinated coffee	Natural: All types of caffeinated coffee	From <once per month to >5 per day	15 months	-	Steatosis development was unaffected by coffee drinking; however, the cross-sectional group showed an inverse association between coffee consumption and liver fibrosis.

ALT—alanine transaminase; AST—aspartate transaminase; CR—calorie restricted; GGT—gamma-glutamyl transferase; GTE—green tea extract; HOMA-IR—Homeostatic Model Assessment of Insulin Resistance; IR—insulin resistance; MD—Mediterranean diet; NAFLD—non-alcoholic fatty liver disease; NASH—non-alcoholic steatohepatitis; n-3 PUFA—omega-3 polyunsaturated fatty acids; UDCA—ursodeoxycholic acid; T2DM—type 2 diabetes mellitus; FGE—garlic extract; FMD—flow-mediated dilation; NOX2—NADPH oxidase 2; FLD—fatty liver disease; GC—green cardamom; hs-CRP—high-sensitivity C-reactive protein; TNF-α—tumor necrosis factor alpha.

**Table 4:** Summarized data of animal studies.

Study	Year	Sample Size	Age	Gender	Intervention	Form of Intervention (Natural or Synthetic)	Dose/Day	Duration	Control	Status after Intervention
Ni X et al. [62]	2016	30 (in 3 groups)	7 weeks	Male C57BL/6J mice	Obeticholic acid or silymarin	Synthetic	Obeticholic acid (30 mg/kg); silymarin (30 mg/kg)	4 weeks	Naïve control group: vehicle (1% HPMC + 1% Tween 20) Vehicle group: vehicle (1% HPMC + 1% Tween 20)	Silymarin significantly reduces liver steatosis.
Meng-sha T. et al. [63]	2021	30 (in 5 groups)	Not mentioned	Male Wistar rats	Silymarin	Synthetic	Silymarin (200 mg/kg) /silymarin (400 mg/kg)	8 weeks	Normal control group: standard chow + water Silymarin control group: water + silymarin (400 mg/kg/day) Fructose control group: 20% fructose solution	Treatment with a high dose of silymarin improves liver function and ameliorates steatosis.
Sun R. et al. [64]	2020	30 (in 5 groups)	6–8 weeks	Male C57BL/6J mice	Silybin + sodium tauroursodeoxycholate	Synthetic	Silybin (50 or 100 mg/kg/day)/ sodium tauroursodeoxycholate (50 mg/kg/day)	4 weeks	Vehicle group: standard normal diet HFD group: high-fat/high-cholesterol diet	Silybin and sodium tauroursodeoxycholate treatment both reduced hepatic lipid accumulation.
Zhu SY et al. [65]	2018	40 (in 4 groups)	2 months	Male ICR mice	Silybum marianum oil (SMO)	Natural (intragastric)	Silybum marianum oil 5 mL/kg Silybum marianum oil 10 mL/kg	8 weeks	Control group: normal diet + distilled water HFD group: high-fat diet (70.5% normal diet + 10% lard + 10% yolk powder + 8% sucrose + 1.5% cholesterol) + distilled water	Silybum marianum oil can significantly reduce fat accumulation and improve lipid metabolism.
Ou Q et al. [66]	2018	18 (in 3 groups)	6 weeks old	Male C57BL/6 mice	Silybin	Synthetic: oral	105 mg/kg/day	8 weeks	Control group: methionine/choline-deficient (MCD) diet (DL-methionine (3 g/kg) + choline bitartrate (2 g/kg)) NASH group: methionine/choline-deficient (MCD) diet	Silybin prevents an increase in steatosis, fibrosis, and liver inflammation.
Ye JH et al. [67]	2016	24	7 weeks	Male C57BL/6J mice	Pentoxifylline (PTX)	Synthetic: intraperitoneal	100 mg/kg	8 weeks	Control group: normal diet HFG group: high-fat diet + hyperglycemia (60 kcal% fat)	Pentoxifylline improves liver function and prevents fat accumulation by upregulating fatty acid β-oxidation.
Massart J. et al. [68]	2012	10–12 mice in 4 groups	5 weeks	Male C57BL/6J-ob/ob mice and C57BL/6J-+/+ mice	Pentoxifylline (PTX)	Synthetic: oral	100 mg·kg^−1^·d^−1^	4 days or 3 weeks	Lean and obese untreated mice	Pentoxifylline can exacerbate fatty liver due to the activation of liver lipogenesis.
Saeed A et al. [69]	2021	16 (in 2 groups)	8–10 weeks/10–12 weeks old	C57BL/6J mice Leptinob mutant (JAX ob/ob)	Vitamin A	Synthetic	20 IU/g	12 or 20 weeks	Controls (C57BL/6J) group: chow diet	Treatment with vitamin A may accelerate the progression of the disease because vitamin A accumulates in hepatocytes.
Ipsen DH et al. [70]	2021	60 chow (*n* = 5) baseline euthanasia (*n* = 6) HFD (*n* = 10) (ASA, *n* = 13) (PTX, *n* = 13) (ASA + PTX, *n* = 13)	Not mentioned	Female Hartley guinea pigs	Acetylsalicylic acid/pentoxifylline/acetylsalicylic + pentoxifylline	Synthetic	46.3 and 45.6 mg/d for acetylsalicylic acid and pentoxifylline	8 weeks	Healthy control group: chow diet HFD group: high-fat diet	NASH or liver fibrosis did not improve in guinea pigs administered acetylsalicylic and pentoxifylline alone or in combination.
Oliveira CP et al. [71]	2003	18 (in 3 groups)	Not mentioned	Male Wistar rats	Vitamin C or vitamin E	Synthetic	Vitamin E (200 mg/day) Vitamin C (30 mg/kg/day)	4 weeks	Control group: choline-deficient diet + vehicle	Vitamin E can prevent the development of steatosis, while vitamin C cannot.
Liu CW et al. [72]	2020	32 (Ctrl) group (*n* = 5) NASH groups (*n* = 9) NASH-resv + EX527 group (*n* = 9) NASH-rest (*n* = 9)	8 weeks	C57BL/6 mice	Resveratrol	Synthetic	NASH-resv group (resveratrol (30mg/kg/day))/ NASH-resv + EX527 group (resveratrol and EX527 (1mg/kg/day))	6 weeks	Control group: normal chow NASH groups: normal chow	Chronic treatment with resveratrol significantly improves hepatic steatosis.
Karimian G. et al. [73]	2015	18 (in 2 groups)	5–6 weeks old	C57BL/6JolaHsd male mice	Vitamin E	Synthetic: vitamin E supplements	20 mg/day	1 week	Control groups: choline-deficient L-amino acid or choline-sufficient L-amino acid	After partial hepatectomy, vitamin E can be used to prevent the progression of steatosis.
Presa N. et al. [74]	2019	20 (5 animals per group)	Not mentioned	Male Pemt +/+ and Pemt −/− mice	Vitamin E	Synthetic: vitamin E supplements	133 IU/kg/day (0.5 g/kg)	3 weeks	Control group: semi-synthetic HFD (60% kcal fat)	Vitamin E dietary supplements can be a suitable treatment to prevent the progression of hepatic steatosis to more advanced stages of the disease.
Lee SW et al. [75]	2021	44	12 to 15 weeks old	Male C57BL/6 SMP30 KO mice and wild-type (WT) mice	Vitamin C	Synthetic: vitamin C supplements	1.5 g/L	11 weeks	Wild-type mice: 60% high-fat diet	Chronic vitamin C deficiency due to de novo lipogen suppression can decrease the progression of MAFLD.
Zeng Q. et al. [76]	2020	60	6–8 weeks	Male C57BL/6 mice	Vitamin C	Synthetic	Low-dose vitamin C group (15 mg/kg per day) Medium-dose vitamin C group (30 mg/kg per day) High-dose vitamin C group (90 mg/kg per day)	Prophylactic group: 12 weeks Therapeutic group: 6 weeks	Control group: normal chow or HFD (25 kcal% fat +1.1% cholesterol and 0.5% cholate ad libitum)	Different doses of vitamin C can have different effects on MAFLD.
Milton-Laskibar I. et al. [77]	2021	50	6 weeks	Male Wistar rats	Pterostilbene and resveratrol	Synthetic: powdered diets	Resveratrol30 group (30 mg/kg/d resveratrol) Pterostilbene group (15 mg/kg/d pterostilbene) Pterostilbene group (30 mg/kg/d pterostilbene)	8 weeks	Control group: standard diet HFHF group: high-fat–high-fructose diet	Changes in gut microbiota that cause disease progression are poorly ameliorated by pterostilbene and resveratrol.
Sun M et al. [78]	2021	46 (in 2 groups)	5 weeks old	Male C57BL/6J mice	Cocoa powder	Natural: cocoa powder	80 mg/g	10 weeks	Control group: high-fat diet	Cocoa supplementation improves steatosis.
Reda D. et al. [79]	2023	32	9 weeks	Male albino rats	Cholecalciferol (vitamin D3)	Synthetic	1000 IU/kg BW (3 days per week)	10 weeks	Negative control: standard rat chow Positive group: high-fat diet (20%) + 25% fructose water Vitamin D control: intramuscular vitamin D (1000 IU/kg BW)	Vitamin D can ameliorative lipid levels in liver and serum.
El-Din SH et al. [80]	2014	90	Not mentioned	Male Sprague Dawley rats	Garlic and onion or combined garlic and onion	Natural: onion oil	Garlic (500 mg/kg b.w) Onion (100 mg/kg body b.w.)	8 weeks	Control group: standard chow diet MAFLD group: high-fat diet (25 % fat + 1% cholesterol + 0.25% bile salts) NAFLD group: switched to regular diet	When administered together, garlic and onion significantly reduced liver steatosis compared to when administered separately.
Wang JH et al. [81]	2021	30	6 weeks old	Male C57BL/6j mice	Cynanchum atratum	Synthetic: Cynanchum atratum powder	100, 200 mg/kg/day	6 weeks	Positive control group: metformin Normal group: chow diet HFD group: high-fat diet (60% kcal% fat) + fructose (20% *w/v*, dissolved in distilled water)	Cynanchum atratum can ameliorate fatty liver by enhancing fatty acid oxidation.
Li S. et al. [82]	2014	83	5 weeks	Male Sprague Dawley rats	Ursolic acid	Synthetic	Group 1: 0.125% ursolic acid (low ursolic acid) Group 2: 0.25% ursolic acid (medium ursolic acid) Group 3: 0.5% ursolic acid (high ursolic acid)	6 weeks	Control group: normal-fat diet HFD group: high-fat diet	Ursolic acid effectively improves hepatic steatosis.
Wang Q. et al. [83]	2020	30 (in 5 groups)/ 15 (in 3 groups)	8 weeks old	Male C57BL/6 mice/ NLRP3 −/− mice	Naringenin	Synthetic	50 or 100 mg·kg^−1^·day^−1^	7 days	Control group: chow diet (0.35% (*w*/*w*) methionine + 0.1% (*w*/*w*) choline + 0.5% sodium carboxymethyl cellulose) MCD groups: methionine/choline-deficient diet (0% methionine + 0% choline, 0.5% sodium carboxymethyl cellulose) NGN 100 group: 100 mg·kg^−1^·day^−1^ naringenin	Naringenin has significant effects on the recovery of liver inflammation and liver steatosis.
Zhou J. et al. [84]	2021	25	4 weeks	Male C57BL/6 mice	Matcha green tea	Natural	0.1%, 0.5%, and 1% matcha	6 weeks	Normal chow diet (NCD) A high-fat diet (HFD)	Matcha supplementation ameliorates lipid accumulation.
Chao J. et al. [85]	2014	31	10 weeks	Male C57BL/6 mice	Gallic acid	Synthetic	50 and 100 mg/kg/day	16 weeks	Normal group: normal chow diet HFD group: high-fat diet (5.24 kcal/g, 60% kcal from lard/soybean)	Gallic acid presents as a natural dietary compound capable of ameliorating NAFLD and other metabolic disorders.
Gao X. et al. [86]	2023	50 (in 5 groups)	58 weeks	Male C57BL/6J mice	Lycopene	Synthetic	Low-dose lycopene group (LLY) (20 mg/kg/d lycopene) High-dose lycopene group (HLY) (60 mg/kg/d lycopene)	8 weeks	Normal control group: normal chow diet (10% calories from fat) + saline solution High-fat and high-fructose diet group: HFD (45% calories from fat) + 10% fructose solution Resveratrol positive control group: high-fat diet + 10% fructose solution +50 mg/kg/d resveratrol	Lycopene dietary supplementation significantly prevented the accumulation of fat in liver.
Gu M et al. [87]	2019	21	6 weeks	Female C57BL/6J mice	Betulinic acid	Synthetic	100 mg·kg^−1^·day^−1^	6 weeks	Chow group: 10% of calories derived from fat High-fat group: HF (60% of calories derived from fat)	Betulinic acid supplementation ameliorated hepatic steatosis and inflammation.
Luo D. et al. [88]	2022	32 (6–8 per 4 groups)	3 to 4 weeks	Male C57BL/6J Narl mice	Tianhuang formula	Synthetic	100 mg/kg/day	6 weeks	Model group (vehicle): high-fat diet (60 fat, 20 proteins, 20% carbohydrate) Atorvastatin group: (1.5 mg/kg/day atrovastamin) Control group: (not mentioned)	The Chinese Tianhuang formula improves MAFLD by regulating intestinal microbiota through its hepatoprotective effect.
Acedo SC et al. [89]	2015	20	6 weeks	Swiss male mice	Pentoxifylline	Synthetic: intraperitoneal	100 mg/kg per day	2 weeks	Lean non-treated group: AIN-93 (control diet; 15% kcal of fat) Lean treated mice: AIN-93 (control diet 15% kcal from fat + 100 mg/kg per day of pentoxifylline) Non-treated obese mice: high-fat diet (HFD; 60% kcal from fat)	Pentoxifylline can improve obesity-related NAFLD by reducing liver inflammation and adipose tissue inflammation.
Panchal K et al. [90]	2012	40	8–9 weeks	Male Wistar rats	Quercetin	Synthetic	0.8 g/kg	8 weeks	Corn starch-rich (C) diet group: 68% carbohydrates as polysaccharides + ~0.7% fat High-carbohydrate, high-fat (H) diet group: 68% carbohydrates fructose + sucrose+ ~24% fat from beef tallow	Quercetin is effective against metabolic syndrome symptoms such as liver complications with debilitating steatosis.
Marcolin E. et al. [91]	2012	64 (in 4 groups)	Not mentioned	C57BL/6 mice	Quercetin	Natural	50 mg/kg	2 or 4 weeks	Control group (CO): control diet + vehicle (sodium carboxymethylcellulose 1%) Control + quercetin group (CO + Q): control diet + 50 mg/kg quercetin NASH group (NASH): with methionine/choline-deficient diet + vehicle (sodium carboxymethylcellulose 1%)	Quercetin improves steatohepatitis by affecting proinflammatory and profibrotic gene pathways.
Panchal SK et al. [92]	2011	72 (in 6 groups)	8–9 weeks old	Male Wistar rats	Rutin	Synthetic	1.6 g/kg food	8 weeks	Corn starch-rich diet group: 68% carbohydrates + 0.7% fat (2 groups) High-carbohydrate, high-fat diet group: 50% carbohydrate (mainly fructose) + 24% fat (mainly beef tallow) + 25% fructose in drinking water (total 68% carbohydrate (2 groups)	Rutin reduces fat deposition in hepatocytes.
Suguro R. et al. [93]	2019	64 (in 8 groups)	Not mentioned	Male wild-type (WT) C57Bl/6 mice	Silybin (SB) and tangeretin (TG)	Synthetic	1.SB (300 or 150 mg/kg) 2.TG (150 mg/kg) 3.SB + TG at low dose (each 75 mg/kg) 4.SB + TG at middle dose (each 150 mg/kg) 5.SB + TG at high dose (each 300 mg/kg)	8 weeks	Control group: vehicle (35% PEG400: 15% Cremophor EL: 5% ethanol: 45% saline) Model group: vehicle (35% PEG400: 15% Cremophor EL: 5% ethanol: 45% saline)	Tangeretin and silybin co-treatment can inhibit de novo synthesis of fatty acids, thus improving lipid profiles.
Kuzu N. et al. [94]	2008	21	Not mentioned	Male Sprague Dawley rats	Epigallocatechin gallate (EGCG)	Synthetic	1 g/L epigallocatechin gallate in drinking water	6 weeks	1. Standard rat diet 2. High-fat diet (HFD)	Epigallocatechin gallate significantly attenuates intrahepatic fat accumulation, inflammation, and fibrosis.
Mao QQ et al. [95]	2021	140 (in 14 groups)	8 weeks	Male C57BL/6J mice	12 types of teas	Synthetic: intragastric	200 mg/kg	15 weeks	Control group: normal diet (3.6 kcal/g, 12% calories from fat) HFD model group: high-fat diet (5.0 kcal/g, 60% calories from fat)	Different teas had different effects, some of which reduced triglyceride content in the liver and improved liver steatosis.
Zhou DD et al. [96]	2022	120 (in 12 groups)	8 weeks	Male C57BL/6J mice	10 types of green tea	Synthetic: intragastric	200 mg/kg	15 weeks	Control group: normal diet (12% calories from fat) HFD model group: high-fat diet (60% calories from fat)	In addition to lowering visceral fat buildup and oxidative stress, a number of green teas can also improve lipid profiles and treat hepatic steatosis.
Liu B. et al. [97]	2019	50 (in 5 groups)	8 weeks	SPF-grade C57BL/6N mice (half male and half female)	Raw bowl tea	Natural: intragastric	50 and 100 mg/kg once a day	12 weeks	Normal group: normal foods + drinking water Model group: high-fat diet Bezafibrate group: bezafibrate (100 mg/kg daily)	Lipopolysaccharides can play a role in the accumulation of fat in the liver. Therefore, raw bowl tea can reduce fat accumulation by reducing lipopolysaccharides.
Li HY et al. [98]	2023	40	4 weeks	C57BL/6J male mice	Theabrownin	Natural: intragastric	2300 mg/kg/day	14 weeks	Blank control group: CD group TB control group: CD + TB group NAFLD with obesity group: HFD group	Theabrownin intervention can effectively alleviate intestinal bacterial dysbiosis and strongly prevent hepatic steatosis.
Li B. et al. [99]	2022	80 (in 8 groups)	8 weeks	C57BL/6J male mice	Six types of tea (different black and dark teas)	Synthetic: intragastric	200 mg/kg b.w./d	15 weeks	Control group: normal diet (3.6 kcal/g + 12% calories from fat) Model group (HFD group): high-fat diet (HFD, 5.0 kcal/g+ 60% calories from fat)	Different extracts of black and dark tea taken as supplements may help prevent aberrant lipid deposition in the liver and liver steatosis.
Torres LF et al. [100]	2019	30 (in 3 groups)	12 weeks	Male C57Bl/6 mice	Green tea	Natural	500 mg/kg b.w.	12 weeks	Control group: chow diet (32% protein, 55% carbohydrates + 13% lipids (2.88 kcal/g)) High-fat diet group: hyperlipidemic diet (20% protein, 36% carbohydrates + 34% lipids (5.31 kcal/g))	Green tea supplementation decreases lipid uptake and accumulation, as well as cholesterol synthesis, through miR-34a and miR-194 modulation in the liver.
Noori M. et al. [101]	2016	18	Not mentioned	Male Sprague Dawley rats	Pomegranate juice	Natural: drinking liquid	60 ± 5 mL/day	7 weeks	Control group: standard chow diet (10% of energy derived from fat + 30% from protein + and 60% from carbohydrates) Model group: high-fat, high-sugar diet (59% of energy derived from fat + 30% from carbohydrates + 11% from protein)	Regular drinking of pomegranate juice can downregulate hepatic proinflammatory and profibrotic conditions and prevent progression to NASH.
El-Lakkany NM et al. [102]	2016	108 (in 9 groups)	Not mentioned	Male Sprague Dawley rats	Metformin and N-acetylcysteine	Synthetic: oral	MTF: 150 mg/kg NAC: 500 mg/kg,	8 weeks	1. Normal control group: standard chow diet 2. NAFLD induced with high-fat diet (HFD): group 25% fat + 1% cholesterol + 0.25% bile salts 3. NAFLD switched to regular diet	In the co-treated group with metformin and N-acetylcysteine, hepatic steatosis significantly decreased, and hepatic fat accumulation reversed.
El-Din SHS et al. [103]	2015	72 (in 9 groups)	Not mentioned	Male Sprague Dawley rats	Rosuvastatin (RSV) and/or β-carotene (βC)	Synthetic: oral	Rosuvastatin: 10 mg/kg/day β-carotene: 70 mg/kg	4 weeks	1. Normal control: standard chow diet 2. NAFLD induced with high-fat diet (HFD) group 3. NAFLD switched to a regular diet group	The combined treatment of rosuvastatin and beta-carotene had a better effect on reducing fat accumulation in the liver than each drug alone.

MAFLD—metabolic dysfunction-associated fatty liver disease; HPMC—hydroxypropyl methylcellulose; HFD—high-fat diet; SMo—Silybum marianum oil; MCD—methionine/choline deficient; NASHA—non-alcoholic steatohepatitis; PTX—pentoxifylline; HFG—high-fat hyperglycemic diet; PEMT—phosphatidylethanolamine N-methyltransferase; WT—wild type; HFHF—high-fat–high-fructose diet; BW—body weight; NCD—normal chow diet; NGN100—naringenin 100; KCs—Kupffer cells; LLy—low-dose lycopene group; HLY—high-dose lycopene group; HF—high fat; AIN-93 diet—American Institute of Nutrition-93 diet; (C) diet—corn starch-rich diet; (H) diet—high-carbohydrate, high-fat diet; CO—control group; CO + Q—control + quercetin group; SB—silybin; TG—tangeretin; EGCG—epigallocatechin gallate; RBTP—raw bowl tea; CD group—blank control group; TB—theabrownin; MTF—metformin; NAC-N—acetylcysteine; RSV—rosuvastatin; βC-β—carotene.

## Data Availability

We used PubMed, SCOPUS, and ScienceDirect databases to screen articles for this review. We did not report any data.
